# Evidence for Underregistration of Suicide

**DOI:** 10.1155/2020/8873893

**Published:** 2020-11-12

**Authors:** M. A. Riedinger, R. F. P. de Winter

**Affiliations:** ^1^Leiden University Medical Centre, Department of Psychiatry, Albinusdreef 2, 2333 ZA Leiden, Netherlands; ^2^Medical Directorate Mental Health Institute Rivierduinen, Sandifoortdreef 19, 2333 ZZ Leiden, Netherlands; ^3^VU Amsterdam, Department of Clinical Psychology, Van der Boechorststraat 1, 1081 BT Amsterdam, Netherlands; ^4^Parnassia Psychiatric Institute, Monsterseweg 93, 2553 R The Hague, Netherlands

## Abstract

In this case report, we will present two cases in which the Dutch municipal coroner registered a natural death, but treating psychiatrists doubted the validity of this decision on the grounds of clinical data and investigation. For both cases, we present evidence that deaths likely resulted from suicide, raising serious doubts about the accuracy of the registered cause of death. According to the WHO bulletin on suicide prevention, the national registration of suicide is unsatisfactory in many countries. The Netherlands is listed by the WHO as having one of the most accurate registration procedures. Nevertheless, there are indications that national registration, even in the Dutch system, is not infallible. In this case report, we present several ways in which the registration process is liable to error and evidence for underregistration of suicide rates.

## 1. Introduction

In 2014, the World Health Organization (WHO) published *Preventing Suicide: A Global Imperative* to raise awareness of a worldwide registered rise in suicides, which they estimate at 800.000 worldwide every year. In this report, the Netherlands is listed as having one of the most accurate registration procedures [1, 2].

In the Netherlands, the cause of death is directly reported to the national bureau of statistics (Centraal Bureau voor Statistiek, CBS) [3]. In 2013, 98.5% of the data matched the registration in the municipal personal records database (Gemeentelijke basis administratie, GBA). A death is always registered by a medical doctor, and after examination of the remains, they note the cause of death. The cause of death is defined as being the concatenation of events that eventually leads to the demise [3]. If the examining doctor is not convinced that it is a death by natural causes, the municipal coroner is asked to examine the remains. Through the public prosecutor, the coroner can instate a formal investigation into the events that led to the demise or deem the death to be of natural causes [4].

Despite the expertise of medical doctors and the reliable route of registration, doubts may remain about the cause of death. In this case report, we will present two cases in which the municipal coroner registered a natural death, but treating psychiatrists doubted the validity of this decision on the grounds of clinical data.

These cases raised the question of whether other psychiatrists have cases of suspected suicide which were deemed to be a natural death. A literature search was performed to ascertain whether similar cases of suicide misregistration have been reported and to identify systemic factors that might contribute to underreporting.

For the literature search in Google Scholar and PubMed, the following strategy was used: “coroners and medical examiners”[MeSH Terms] OR (“coroners”[All Fields] AND “medical”[All Fields] AND “examiners”[All Fields]) OR “coroners and medical examiners”[All Fields] OR “coroner”[All Fields]) AND (“suicide”[MeSH Terms] OR “suicide”[All Fields]), registration [All Fields] AND (“suicide”[MeSH Terms] OR “suicide”[All Fields] OR “suicides”[All Fields]) AND “reliable” [All Fields] OR “suicide and registration” [All Fields].

## 2. Case Presentations

### 2.1. Case 1

A physically healthy woman in her 50s was admitted to the closed psychiatric ward due to suicidal thoughts. The preliminary diagnosis was an acute episode of depression due to psychosocial stressors and diagnosed with an adjustment disorder. Precipitant stressors included the hospitalization of a chronically ill partner with whom she had long had a contentious relationship.

She had been getting reproachful remarks from him for weeks, and he had been prescribed quetiapine because of aggressive behavior. She denied alcohol or substance abuse and admitted there had been problems in the marriage for some time.

She had been sleeping badly for a number of weeks. Because of the lack of effect of treatment with a benzodiazepine, and the probable association of insomnia with rumination and suicidal thoughts, a low dosage of an atypical antipsychotic medicine (quetiapine 50 mg) was prescribed, with a reasonable effect on the sleeping quality. After a week, the symptoms were attenuated enough to permit a leave of two days during the weekend, in which she was to be telephoned by the nurses of the psychiatric ward. She was considered to be in phase 2 of a structured suicide risk management taxonomy [5]. When the nurses could not establish contact with this patient, she was actively searched for. Her remains were found in her house, and the municipal coroner determined that unilateral pneumonia was the cause of death. The treating psychiatrist suspected suicide and contacted the coroner, but the municipal coroner did not instigate further investigation.

The conclusion that a healthy woman would die of one-sided pneumonia without earlier symptoms is deemed to be very unlikely. In the days following her demise, the treating physicians contacted her close relatives and together decided to request an autopsy, arranged and paid for by the psychiatric institution. During the autopsy, a lethal blood concentration of quetiapine in combination with ethanol was found. The psychiatric team and the pathologist were convinced that her demise was caused by an intentional overdose in combination with alcohol.

This combination probably resulted in aspiration and suffocation. However, in official statistics, the cause of death was never adjusted, and this suicide was, in our opinion, falsely registered as a natural death.

### 2.2. Case 2

A man in his 50s was admitted to the closed psychiatric ward because of suicidal thoughts and stress, due to complicated psychosocial circumstances. He started in phase 4 [1–5] of a structured suicide risk management taxonomy [5], and after a day, this was decreased to phase 3. There were no somatic problems.

The patient history encompassed a major depressive and personality disorder, with several serious suicide attempts, including overdoses with pharmaceuticals, for which he had been admitted to the intensive care unit before. The cause of the present suicidal thoughts upon admission was a serious allegation from a close family member. The partner of the patient had ended the relationship due to these allegations and filed for divorce. There were also work-related problems and debts. Because of the high estimated risk of suicide, the patient stayed at the closed ward for several weeks. In addition, the tricyclic antidepressant medication (TCA) was increased. The dosage was near its maximum, resulting in therapeutic blood levels. Hereafter, the patient denied having any remaining suicidal thoughts, and after some days, in phase 2 [1–5] of a structured suicide risk management taxonomy [5], he was discharged to a home that had been found for him with the help of social services. Two days after being discharged, the patient was found dead in his home. The municipal coroner registered a natural death by heart attack, and no autopsy was commissioned. Through a family member, treating physicians learned that during the tidying of the house, around 5 empty medication strips (containing 10 tablets each) of the tricyclic antidepressant were found. This specific medication is lethal in higher dosages, and an overdose can cause adverse cardiac effects. Because this drug has a relatively narrow therapeutic window and there was no history of cardiac problems, cardiac arrest as a result of an overdose was deemed to be a likely cause of death. Additionally, no other medication could be found in the house, even though the patient had recently filled his prescriptions at his pharmacy. Upon discharge, he had been given a prescription for 2 weeks with separate medication for 3 days. It was later learned that he had also been to the general practitioner for a prescription. Considering the patient's recent admission into a psychiatric ward due to suicidal thoughts, the previous serious suicide attempts, and the physical health of this patient, we highly suspect this case to be death by suicide. However, no further postmortem investigation took place and it was not recorded as such in official records.

## 3. Discussion

There are many ways in which the registration of the cause of death can become flawed. Overreporting of suicides can happen for many reasons, for example, an inadequate investigation into a possible murder, or lethal accidents involving psychiatric patients or people with a history of suicidal ideation. Also, some deaths are ruled suicides, in which close relatives or friends have reasons to believe the actual cause of death was murder. Thus theoretically, overreporting could be a reason why suicide statistics are not completely accurate. However, in the literature, overreporting does not seem to structurally influence statistics on cause of death [6, 7].

Underreporting of suicides on the other hand, of which we suspect the presented cases to be examples, seems to be the case more often than overreporting. It happens worldwide, and for a multitude of reasons, several of which are listed below [1, 8–11].

Firstly, of the 172 member states of the WHO, only 60 have a satisfactory method for registering cause of death, leading them to have relatively reliable statistics on suicide. The Dutch method of registration is counted among the best, but worldwide, many causes of death are registered as “accidents” or “not otherwise specified.” When Dutch citizens die in countries with a less organized healthcare system, the cause of death is often less reliably investigated. A lethal accident, risky behavior or the ingestion of drugs, could be premeditated and based on suicidal ideation [12]. Some cases of missing persons could also be caused by suicides in which the remains are never discovered.

Secondly, suicide is a taboo in many cultures and therefore not registered in some countries. One study showed that in 35 of the 192 investigated countries, suicide and suicide attempts are still punishable. In 25 countries, they are punishable by law, and in a further 10 countries, people can be tried for this under Sharia law [1, 13]. Because there is such a taboo on psychiatric disorders and any form of suicidal ideation in some countries, registration of these problems in medical dossiers is often waived or adjusted to a less shameful diagnosis.

Thirdly, in some countries, medical costs are not reimbursed if the cause for treatment was a suicide attempt. It is therefore probable that deaths resulting from sustained injuries from suicidal behavior are not registered as suicides due to financial considerations [13, 14].

Fourthly, even when the cause of death is determined by a trained municipal coroner, human mistakes are still a liability in the quality of registration.

Fifthly, studies that investigated the interobserver variability between coroners after an autopsy showed that coroners are more inclined to register the cause of death as accidental when it concerns cases of drug overdosage or drowning, even when many facts point towards a suicide [11, 15, 16].

Lastly, the amount of proof different professionals need in order to be certain of a suicide and register it differs. Even though the method of registration does not differ much, the amount of information on a death certificate can vary widely. In two comparative studies by Tøleffsen et al. [17, 18], the causes of death were reevaluated by coroners, psychiatrists, and trained coders. Demises previously registered as suicides were deemed to be accidents after reevaluation, and vice-versa. The changes did not lead to a statistical difference in the number of suicides; therefore, these studies illustrate how complex it can be to determine the cause of death.

Ruling a death to be a suicide requires an insight into the motives behind the actions just before death [19]. Only in 20-43% of suicides, a suicide note was found [20, 21]. In all these studies, authors suggest that more profound investigation into the cause of death, for example, through autopsy, the last days of the deceased, and possibly previous suicidal behavior, may contribute to a more accurate way of determining cause of death. A meta-analysis by Arsenault-Lapierre et al. [22] in 2004 showed that 87.3% of the people that die due to suicide have a history of psychiatric disease. However, this fact is not the holy grail to recognizing a suicide, since only 40% of suicide victims in the Netherlands ever come into contact with mental healthcare providers [23].

It is noteworthy that 6126 cases of euthanasia or physician-assisted deaths took place in the Netherlands in 2018, of which 67 listed psychiatric disorders as the reason [24]. It is important to note that these deaths are registered as natural deaths.

## 4. Conclusion

In the WHO report, it is estimated that each year, 800.000 people die of suicide worldwide. However, even leading countries like the Netherlands have difficulties correctly registering suicides. Both of the cases in this paper illustrate the challenges that the registering doctors are faced with when registering a suicide and demonstrate that suicide statistics are not completely reliable [1, 25]. Since many victims of suicide have a history of psychiatric disorders, we would like to suggest consulting a psychiatrist, a municipal coroner, or commissioning an autopsy in case of a demise which leaves even the slightest trace of doubt about the cause of death. In the Netherlands, an autopsy can even be financed through public health insurance and commissioned by the treating psychiatrists or family of the demised.

We call on colleagues worldwide to stay alert and report possible wrongly registered deaths to us, to make a broader study possible.
